# Propolis-Based Solution Application in Extraction Socket Healing: A 3D Split-Mouth Clinical Pilot Study

**DOI:** 10.7759/cureus.112041

**Published:** 2026-07-04

**Authors:** Georges E Moussallem, Ronald Younes

**Affiliations:** 1 Faculty of Dental Medicine, Saint Joseph University of Beirut, Beirut, LBN; 2 Department of Oral Surgery, Faculty of Dental Medicine, Saint Joseph University of Beirut, Beirut, LBN

**Keywords:** 3d intraoral scanning, natural adjunct, oral surgery, pain management, propolis, socket healing, soft-tissue healing, split-mouth study, tooth extraction, wound healing

## Abstract

Background

Propolis is a natural resinous substance produced by honey bees that possesses well-documented anti-inflammatory, antimicrobial, antioxidant, and tissue-regenerative properties. Despite extensive investigation in dermatological wound healing, high-quality clinical evidence regarding its role in promoting oral soft-tissue healing following tooth extraction remains limited.

Aim

This study aimed to evaluate whether a commercial formulation containing propolis extract, clove oil, and white willow bark enhances soft-tissue healing following tooth extraction compared with standard postoperative care.

Material and methods

A prospective split-mouth controlled pilot study was conducted between September 2025 and April 2026. Thirteen patients requiring bilateral tooth extractions of similar surgical difficulty were recruited. Both extraction sites received identical postoperative care with saline mouthwash; the test site additionally received topical application of the commercial propolis-containing formulation. Soft-tissue healing was assessed using intraoral 3D scans at baseline (D0), day 7 (D7), and day 14 (D14), with surface area closure calculated as a percentage of baseline socket area. Postoperative pain was evaluated using a visual analog scale (VAS) on days 1, 3, and 7. Linear mixed-effects models were used for inferential analysis.

Results

Twenty-six extraction sockets from 13 patients were analyzed at D7, and 24 extraction sockets from 12 patients were analyzed at D14 following one loss to follow-up. Socket closure increased significantly between D7 and D14 in both groups (p < 0.001). A significant main effect of treatment group was observed (p = 0.011), demonstrating greater overall socket closure in propolis-treated sites compared with control sites. Postoperative pain decreased significantly over time in both groups (p < 0.001) but did not differ significantly between groups (p = 0.879).

Conclusion

The tested propolis-containing commercial formulation was associated with greater soft-tissue closure following dental extraction. Further well-designed studies with larger sample sizes are needed to confirm these findings.

## Introduction

Soft-tissue healing following tooth extraction is essential for uneventful postoperative recovery and the prevention of local complications, such as infection, delayed epithelialization, and patient discomfort. While the natural healing process leads to satisfactory closure of the socket, the rate of epithelialization can vary depending on local and systemic factors. Enhancing soft-tissue repair while minimizing inflammation and microbial contamination remains an important objective in the field of oral surgery.

In recent years, growing interest has been directed toward natural bioactive agents capable of enhancing wound healing without the drawbacks associated with synthetic medications. Propolis, a resinous material produced by honey bees from plant exudates, has demonstrated well-documented antimicrobial, anti-inflammatory, antioxidant, and regenerative properties. These biological effects have been attributed primarily to its rich content of flavonoids, phenolic acids, and aromatic esters, which collectively promote epithelial regeneration and modulate the inflammatory response. Although propolis has been extensively studied in dermatological and medical wound applications, its direct role in accelerating oral soft-tissue healing remains under-investigated.

The present study was designed to evaluate the efficacy of propolis in enhancing the rate and quality of mucosal healing following tooth extraction. By assessing clinical healing parameters and patient outcomes, this research seeks to evaluate whether the tested formulation may serve as a natural, biocompatible, and cost-effective adjunct to promote faster soft-tissue regeneration in the oral cavity.

The null hypothesis was that the use of propolis does not produce any significant improvement in the rate or quality of mucosal healing following tooth extraction compared to standard care, as assessed by clinical healing parameters and patient-reported outcomes.

Dental extractions remain one of the most frequently performed procedures in maxillofacial and oral surgery. Post-extraction sockets represent open wounds exposed to the microbe-rich oral environment of the oral cavity, thereby increasing susceptibility to bacterial contamination, inflammation, and mechanical disruption. Complete physiological repair relies heavily on efficient soft-tissue repair, which requires the stability and formation of a blood clot, as well as minimal bacterial contamination. Adequate and rapid epithelial coverage is crucial for restoring the mucosal barrier and preventing any prolonged inflammation of both soft and hard tissues (osteitis) [1,2].

Delayed soft-tissue healing has been associated with postprocedural pain, inflammation, and an increased risk of local complications. These physiological processes are particularly relevant to immunocompromised populations such as those with periodontitis, diabetes mellitus, or bisphosphonate-associated syndromes [3].

Biological phases of socket healing (soft-tissue perspective)

Subsequent to extraction procedures, the alveolar socket healing follows a chain of overlapping biological stages, with the hemostasis phase forming prior to the inflammation phase. Hemostasis occurs once blood clot formation is stabilized, serving as a provisional matrix and providing the essential inflammatory mediators to initiate proper tissue repair [1].

Inflammatory cells, such as neutrophils and macrophages, initiate the inflammation phase, playing a crucial role in host defense against microbial pathogens and debris clearance with the release of cytokines and growth factors that modulate the healing mechanism and dominate the inflammatory phase [4].

During the proliferative phase, fibroblast proliferation occurs along with angiogenesis and granulation tissue formation. Concomitantly, epithelial cells migrate from the wound margins to cover the socket surface, leading to the progressive closure of the epithelium [3]. This phase represents a critical stage of soft-tissue healing and is the primary target for therapeutic modulation.

Clinical examination of soft-tissue healing post extraction

Clinical assessment of soft-tissue healing following surgical procedures includes parameters such as tissue color, edema levels, bleeding tendency, epithelial border continuity, and presence of inflammation or granulation exudate. Standardized indices for evaluation have been adapted in the literature for decades, with an evolution aiming to minimize subjectivity and improve reproducibility [5]. While being the standard evaluation protocol, conventional techniques may lack accuracy in detecting and classifying physical changes in soft-tissue healing. As a result, there is growing interest in integrating digital and image-based methods to complement clinical evaluation and provide more objective and reproducible measurements of soft-tissue closure [6].

Emerging strategies in enhancing post-extraction tissue healing

Several treatment modalities have been explored with the aim of aiding and accelerating soft-tissue healing following tooth extractions, such as sutures, antiseptic and antimicrobial agents, and topical wound dressings. Conventional postoperative agents such as chlorhexidine and povidone iodine have been used for decades, with numerous studies supporting their properties in promoting faster healing [7]. Although beneficial, no approach is without limitation, thus promoting the exploration of adjunctive therapies with superior therapeutic outcomes. Hyaluronic acid and herbal-derived products have been investigated for their immunomodulatory potential and the promotion of epithelialization.

Propolis in oral soft-tissue healing

Propolis has been proven to be a promising biomaterial owing to its antimicrobial, anti-inflammatory, and wound-healing capacities, with broad-spectrum antimicrobial activity against common pathogens [8]. Studies regarding propolis have highlighted its immunomodulatory role by inhibiting pro-inflammatory mediators and limiting oxidative stress, thereby promoting favorable conditions for healthy tissue repair [9]. Experimental studies have reported enhanced fibroblast activity, increased collagen deposition, and accelerated epithelialization in studies involving the topical application of propolis solution in oral soft tissue, as well as bone regeneration advantages.

Evidence gaps in current literature

Although the current literature has extensively explored post-extraction healing, the majority of studies focus on alveolar bone changes and ridge or socket preservation techniques, thereby lacking soft-tissue-focused outcomes. There remains a paucity of clinical studies evaluating adjunctive therapies aimed at supporting soft-tissue regeneration, more specifically in post-extraction situations. Furthermore, limited clinical evidence has been acquired regarding the efficiency of propolis in the healing of oral tissue and its promotion as a standard product of care for oral wounds.

Background and objective of the study

Given the importance of soft-tissue healing in the oral mucosa and extraction sites and the promising capacities of propolis usage, further research is warranted. The present split-mouth clinical study was designed to evaluate the effect of direct product application on dental sockets using standardized and digital assessment techniques, highlighting accurate and objective measurements of the investigated wound sites.

## Materials and methods

Study design and protocol

A controlled split-mouth study was conducted between September 2025 and April 2026. Fourteen patients were assessed for eligibility, and 13 patients were finally included. All participants were selected in compliance with the eligibility criteria. The study followed a prospective and split-mouth design, with each patient contributing two comparable extraction sockets, permitting within-subject comparison and reducing between-subject variability. All extractions were performed during the same session by the same operator, using an identical surgical protocol for both sites.

Ethics consideration

The present clinical study was conducted in strict accordance with the ethical principles outlined in the Declaration of Helsinki and complied with the Consolidated Standards of Reporting Trials (CONSORT) guidelines for clinical research reporting [10]. Ethical clearance was obtained from the Clinical Research Ethics Committee of Saint Joseph University of Beirut prior to study initiation (No. Tfemd-2026-54). All participants were thoroughly informed about the study’s objectives, methodology, and potential implications, and written informed consent was obtained before inclusion. This study was undertaken at a single academic center, namely, the Faculty of Dental Medicine, Medical Sciences Campus, Saint Joseph University. All collected data were de-identified and systematically coded in full compliance with the Health Insurance Portability and Accountability Act (HIPAA) regulations. This trial was registered under ClinicalTrials.gov (identifier: NCT07665814).

Sample size calculation

For a paired split-mouth design, assuming a moderate paired effect size, an alpha level of 0.05, and 80% power, the estimated sample size was approximately 23 patients using G*Power 3.1 software (Heinrich Heine University Düsseldorf, Düsseldorf, Germany). During the study period, 14 patients were assessed for eligibility, and 13 patients were finally included, each contributing one test site and one control site. The final sample was therefore lower than the estimated sample size and should be considered when interpreting the findings.

Inclusion and exclusion criteria

The inclusion criteria required: (a) cooperative adult patients accepting of the protocol, (b) cooperative parent/legal guardian consenting for participating minors, (c) non-surgical extractions of bilaterally positioned teeth, (d) low-to-moderate difficulty extraction according to the Pederson scale, and (e) healthy patients with no history of systemic disease.

The exclusion criteria included: (a) non-consenting patients, (b) failure to attend follow-up appointments per protocol, (c) heavy smokers (more than 10 pack-years), (d) systemic disease such as diabetes mellitus or immunosuppressive syndromes, (e) patients under contraceptive medication, (f) patients under systemic antibiotic therapy for the last three months or anti-coagulants in the previous four-week period, (g) patients requiring antibiotic prophylaxis, (h) pregnant or breastfeeding women, (i) patients with a known history of honey or bee-related allergens, (j) patients with a history of allergies to local anesthetic, and (k) higher-difficulty extractions requiring sutures or other hemostasis measures.

Randomization and allocation

**Figure 1:**
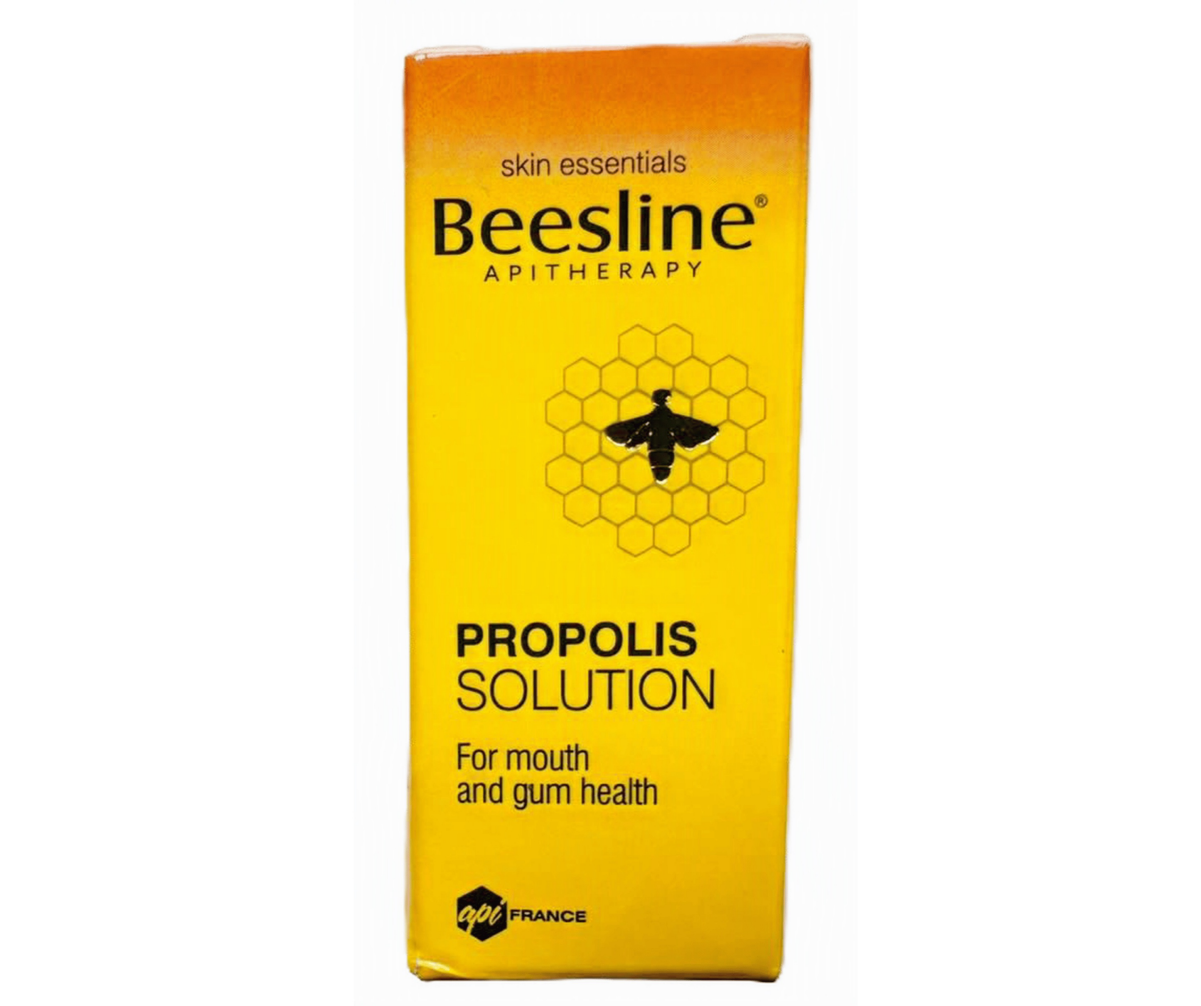
Beesline propolis solution. Photograph of Beesline propolis (Beesline International S.A.L.; Sin El Fil, Beirut, Lebanon) obtained using a Canon EOS R100 digital camera with an RF 100 mm F2.8 L Macro IS USM lens (Canon Inc., Tokyo, Japan).

The propolis solution was provided by Beesline International S.A.L. (Sin El Fil, Beirut, Lebanon), contained in a 5 mL amber glass bottle with an integrated brush applicator (Figure 1). The formulation contained the following ingredients: propolis extract 52% as the active ingredient; denatured alcohol (Alcohol Denat.) serving as solvent and carrier medium; *Eugenia caryophyllus* (clove) bud oil acting as an antiseptic and analgesic agent; and *Salix alba* (willow) bark extract included for its anti-inflammatory and soothing properties.

**Figure 2:**
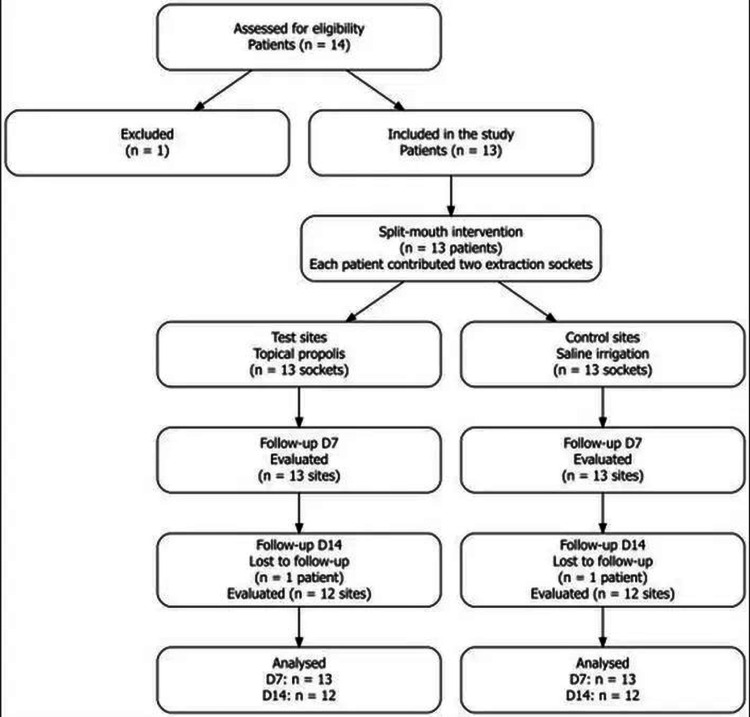
Flowchart of patient enrollment. Generated by RStudio Version 2026.04.0 Build 525 (Posit PBC, Boston, MA).

Detailed information regarding the concentration, composition, and component ratios of the formulation was not provided by the manufacturer. Once patients were selected as eligible to participate in the study, the application site was assigned, and patients received explicit instructions regarding product application to ensure standardization (Figure 2). Randomization of the test and control extraction sites within each participant was performed using a coin-flip procedure conducted by the treating operator immediately prior to the intervention. Allocation was not concealed, as the operator was aware of the outcome at the time of treatment; however, outcome measurements were performed by a blinded, independent examiner (see Data collection).

Interventions and instructions

All extractions were performed concurrently in a single operative session by the same operator as part of the split-mouth study design. All patients underwent a preoperative clinical examination and panoramic radiography (orthopantomogram) using the VistaPano S (Dürr Dental SE, Bietigheim-Bissingen, Germany) to evaluate the maxillomandibular structures and confirm eligibility. Surgical procedures were performed under local anesthesia with 4% articaine and 1:200,000 epinephrine. Standard protocol for non-surgical extractions was adapted, including gingival fiber detachment (syndesmotomy), dental luxation, alveolar socket expansion, forceps-assisted tooth delivery, socket inspection, and hemostasis.

Once the extractions were completed, patients were instructed to rinse with saline mouthwash and apply the topical propolis solution three times per day on the designated extraction test site, starting the day after extraction for seven days until the first follow-up (D7) appointment. Patients were instructed to brush their teeth and rinse with saline prior to application and to dry the area with a sterile gauze before administration to enhance product adhesion and retention.

All participants were prescribed paracetamol (acetaminophen) 500-1000 mg every four to six hours for adults and adolescents for the first day post-extraction, and for the following days if necessary. A standard visual analog scale (VAS) was used to measure pain scores for each patient on postoperative days 1 (D1), 3 (D3), and 7 (D7), with patients reporting back to the operator via online communication. Patient compliance with the prescribed application regimen was monitored by recollecting the propolis bottles on day 7 and visually confirming that the product had been used.

Clinical examination and data acquisition

Examinations for all cases were performed by a single trained, blinded examiner. Intraoral scans were obtained preoperatively, postoperatively at day 0 immediately post-extraction (D0), day 7 (D7), and day 14 (D14) using a Medit intraoral scanner i700 (Medit Co., Ltd., Seoul, South Korea) and Medit Link scanning software. The standard polygon format files (PLY) were imported into mesh analysis software for three-dimensional evaluation. Serial scans were aligned using stable, unchanged anatomical reference areas to ensure accurate registration.

Intraoral scanning protocol

Prior to scanning, the surgical site was gently irrigated and wiped using sterile gauze to ensure optimal moisture control and prevent stitching artifacts. Patients were positioned in a semi-supine position. The customized scanning protocol involved: (1) a preliminary scan before extraction as baseline; (2) locking of the surface data as reference; (3) polyline trimming of the extraction site perimeter using 4 securing limit points; (4) a scan of the socket immediately post-extraction; (5) cloning the model and scanning again on D7; (6) repeating the same sequence on D14. All scans were reviewed immediately after acquisition, and datasets were exported in PLY format without mesh smoothing to preserve raw geometric data integrity.

Digital measurement protocol

**Figure 3:**
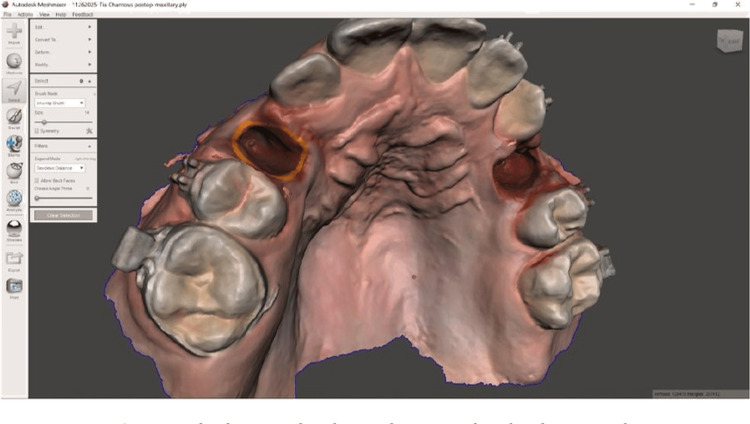
Manual selection of the socket surface using the brush selection technique. Generated using Autodesk Meshmixer (version 3.5.474; Autodesk Inc., San Francisco, CA).

**Figure 4:**
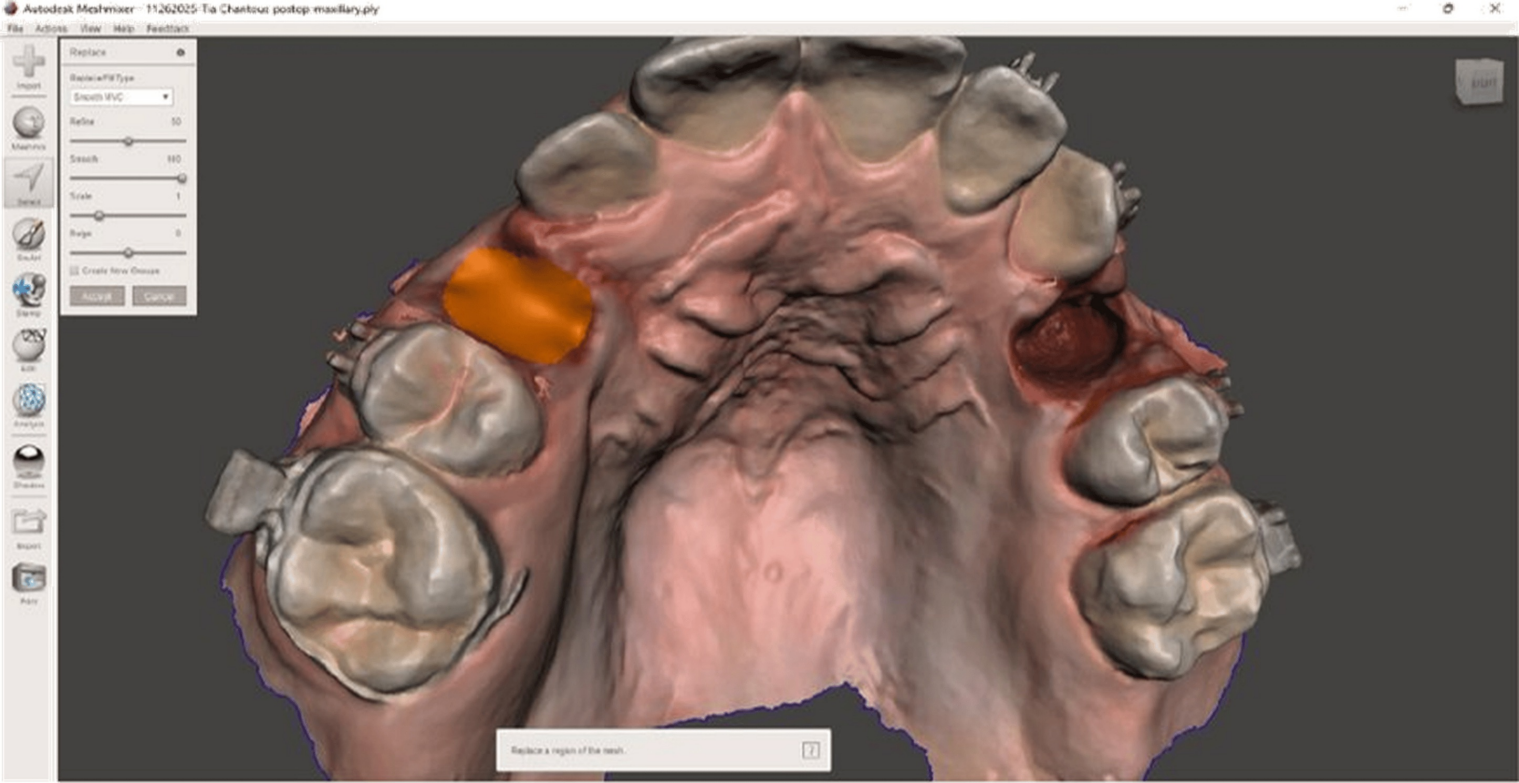
Surface area replaced by Smooth Mean Value Coordinates (MVC) setting. Generated using Autodesk Meshmixer (version 3.5.474; Autodesk Inc., San Francisco, CA).

**Figure 5:**
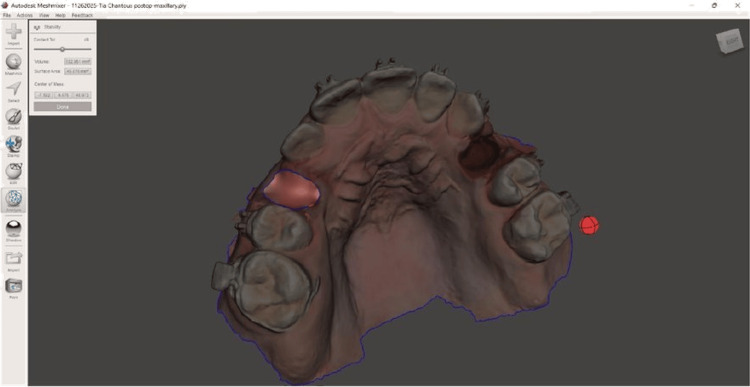
Surface area analysis using the Stability tool. Generated using Autodesk Meshmixer (version 3.5.474; Autodesk Inc., San Francisco, CA).

All PLY datasets were imported into Autodesk Meshmixer (version 3.5.474; Autodesk Inc., San Francisco, CA) for three-dimensional inspection. Digital models were aligned using a surface-based registration protocol, with initial manual alignment, followed by automatic registration based on stable reference structures. After registration, the socket perimeter was manually delineated on the baseline model (D0) using the standardized brush selection technique (Figure 3). The surface area was then flattened using the flat mesh option (Figure 4), and the region of interest (ROI) was isolated for accurate surface area analysis (Figure 5).

For each given scan model, surface area changes were calculated to determine the percentage of soft-tissue closure between timestamps using the following formula:

$\% \text{ Reduction} = \frac{A_{\mathrm{baseline}} - A_{\mathrm{timepoint}}}{A_{\mathrm{baseline}}} \times 100$

Measurements were made for each control and test site at D0, D7, and D14. All measurements were performed twice by the same examiner to reduce variability.

Statistical analysis

All statistical analyses were performed using RStudio (version 2026.04.0 Build 525; Posit PBC, Boston, MA). Two outcome variables were evaluated separately: soft-tissue healing, assessed by the percentage reduction in extraction socket surface area, and postoperative pain, assessed using VAS scores. Healing was expressed as the percentage of surface area closure relative to baseline (D0). Descriptive statistics included mean ± SD, median, IQR, minimum, and maximum values.

Inferential analyses were performed using linear mixed-effects models. For soft-tissue healing, the model included treatment group, time (D7 vs. D14), and their interaction as fixed effects, with patient ID as a random effect. For postoperative pain, a similar model was fitted including treatment group, time (D1, D3, D7), and their interaction, with patient ID as a random effect. One participant was lost to follow-up prior to the D14 evaluation. The linear mixed-effects models used all available observations at each timepoint, retaining this participant's D7 data; therefore, D7 analyses included 13 patients (26 extraction sockets), and D14 analyses included 12 patients (24 extraction sockets). Type III ANOVA with Satterthwaite-adjusted degrees of freedom was employed. A threshold of p ≤ 0.05 was adopted for statistical significance.

## Results

Soft-tissue healing (surface area closure)

Descriptive Statistics

**Figure 6:**
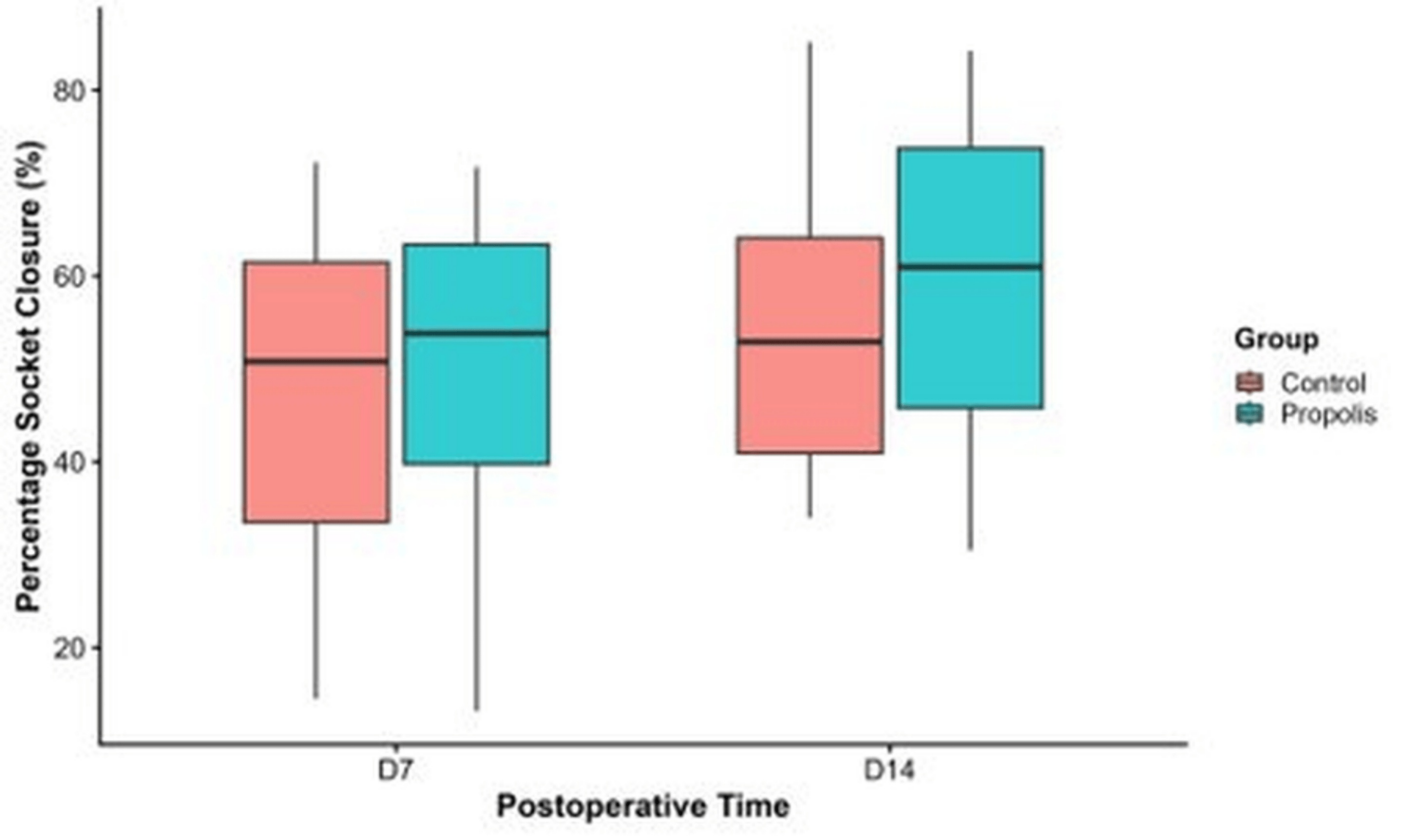
Distribution of percentage socket closure by treatment group at postoperative days 7 and 14. Generated by RStudio Version 2026.04.0 Build 525 (Posit PBC, Boston, MA).

**Figure 7:**
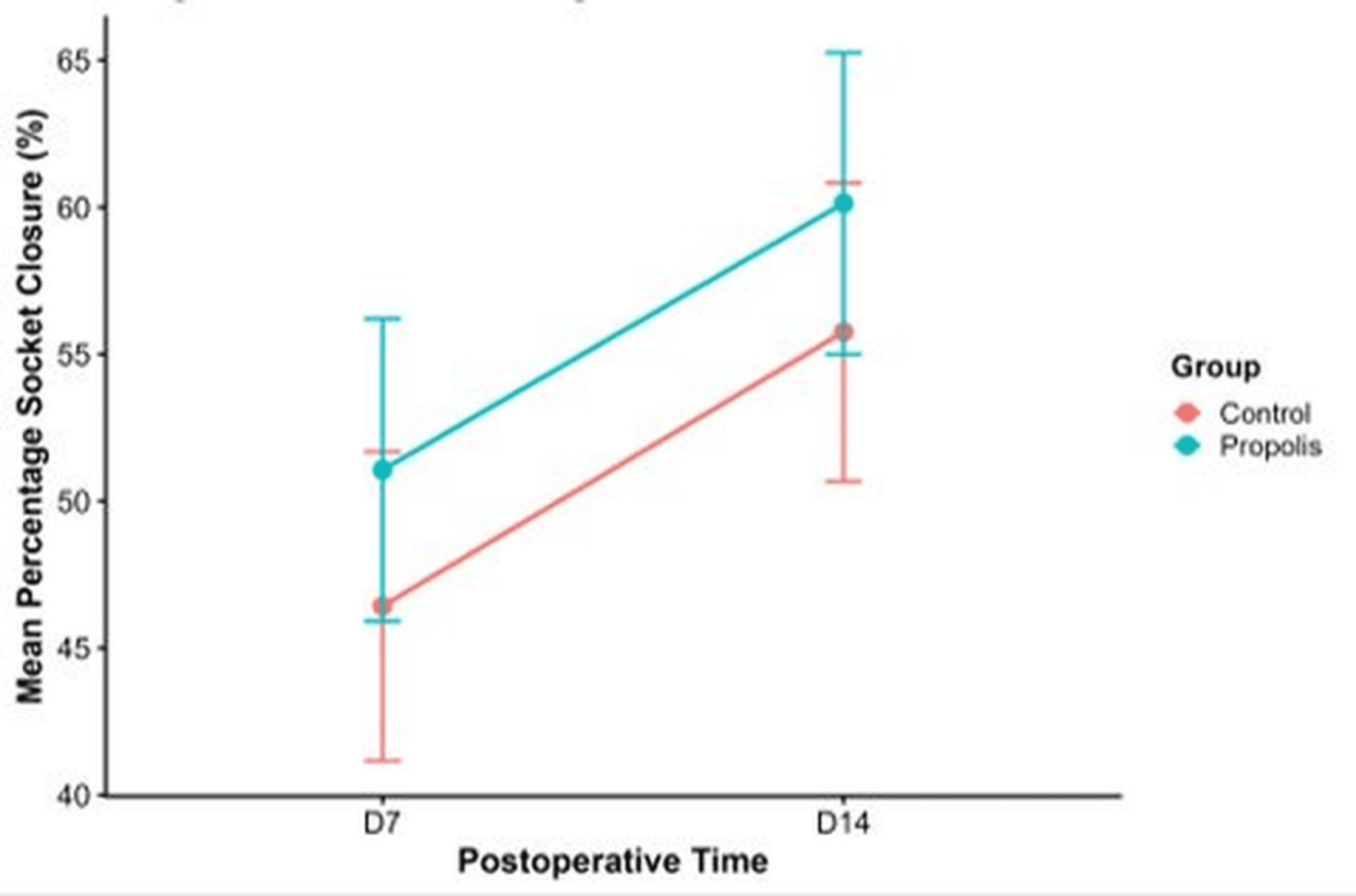
Mean percentage socket closure over time in control and propolis-treated extraction sites. Generated by RStudio Version 2026.04.0 Build 525 (Posit PBC, Boston, MA).

Descriptive statistics for percentage socket closure are presented in Table 1. The percentage of socket closure increased between postoperative D7 and D14 in both treatment groups. At D7, the mean closure was 46.4 ± 19.0% for control sites and 51.1 ± 18.6% for propolis-treated sites. By D14, mean closure increased to 55.8 ± 17.6% in control sites and 60.1 ± 17.8% in propolis-treated sites. Propolis-treated sockets demonstrated higher closure values at both time points; however, inter-individual variability was observed (Figures 6, 7).

**Table 1:** Descriptive statistics for percentage socket closure.

Group	Time	n	Mean ± SD (%)	Median (%)	IQR	Min-Max (%)
Control	D7	13	46.4 ± 19.0	50.8	28.0	14.5-72.3
Control	D14	12	55.8 ± 17.6	52.9	23.2	34.0-85.2
Propolis	D7	13	51.1 ± 18.6	53.8	23.6	13.2-71.7
Propolis	D14	12	60.1 ± 17.8	61.0	27.9	30.5-84.2

Inferential Statistics

A linear mixed-effects model revealed a significant main effect of time (F = 36.20, p < 0.001), indicating that socket closure increased significantly between D7 and D14 in both treatment groups. A significant main effect of treatment group was also observed (F = 7.29, p = 0.011), demonstrating greater overall socket closure in propolis-treated sites compared with control sites. The group × time interaction was not statistically significant (p = 0.938), indicating that the magnitude of improvement between D7 and D14 was similar for both groups. Estimated marginal means are presented in Table 2.

**Table 2:** Estimated marginal means of percentage socket closure.

Group	Time	n	Mean ± SD (%)	Median (%)	IQR	Min-Max (%)
Control	D7	13	46.4 ± 19.0	50.8	28.0	14.5-72.3
Control	D14	12	55.8 ± 17.6	52.9	23.2	34.0-85.2
Propolis	D7	13	51.1 ± 18.6	53.8	23.6	13.2-71.7
Propolis	D14	12	60.1 ± 17.8	61.0	27.9	30.5-84.2

Postoperative pain (VAS)

Descriptive Statistics

**Figure 8:**
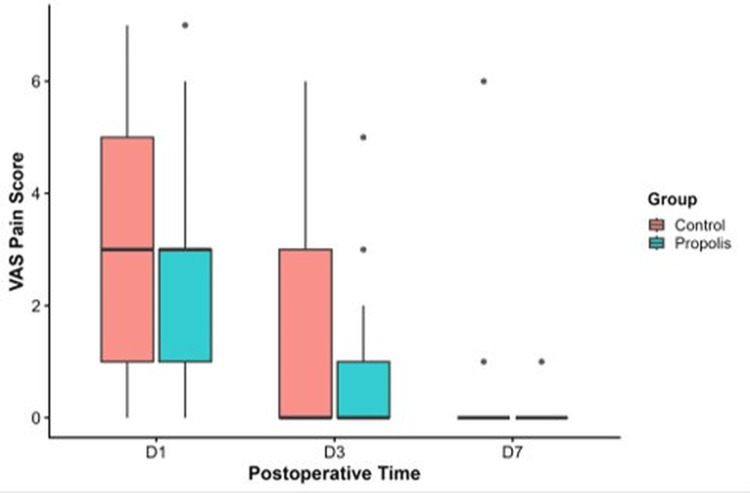
Distribution of postoperative pain scores (VAS) by treatment group at days 1, 3, and 7. VAS: visual analog scale. Generated by RStudio Version 2026.04.0 Build 525 (Posit PBC, Boston, MA).

**Figure 9:**
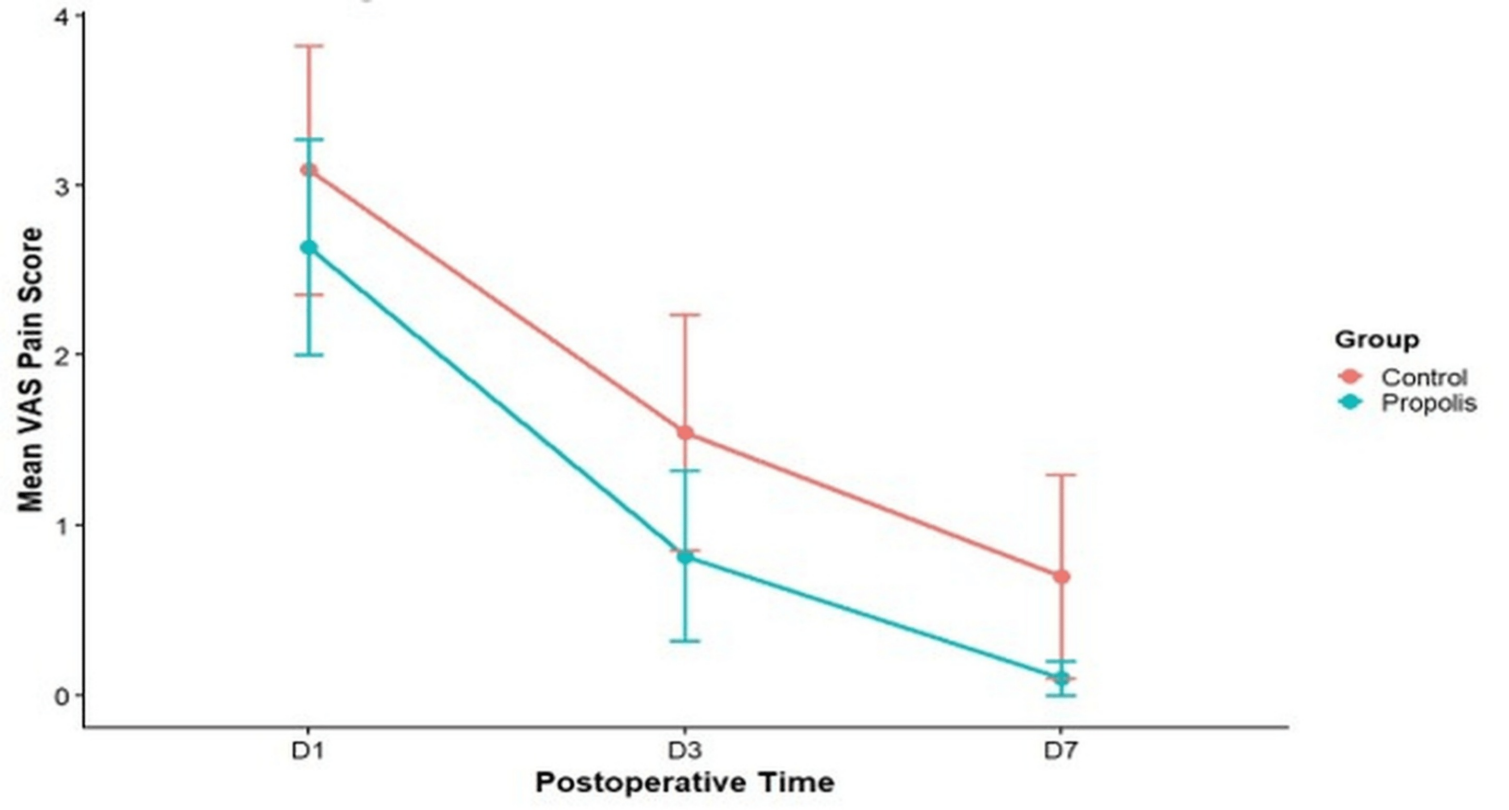
Mean postoperative pain scores (VAS) over time in control and propolis-treated sites. VAS: visual analog scale. Generated by RStudio Version 2026.04.0 Build 525 (Posit PBC, Boston, MA).

VAS pain scores decreased over the postoperative period in both treatment groups (Table 3). At D1, the mean VAS score was 3.08 ± 2.53 in control sites and 2.69 ± 2.29 in propolis-treated sites. At D3, the mean pain scores decreased to 1.62 ± 2.26 and 0.85 ± 1.57, respectively. By D7, pain levels were minimal in both groups, with mean scores of 0.58 ± 1.73 in control sites and 0.08 ± 0.29 in propolis-treated sites (Figures 8, 9).

**Table 3:** Descriptive statistics for visual analog scale (VAS) pain scores.

Time	Control (Mean ± SD)	Propolis (Mean ± SD)
D1	3.08 ± 2.53	2.69 ± 2.29
D3	1.62 ± 2.26	0.85 ± 1.57
D7	0.58 ± 1.73	0.08 ± 0.29

Inferential Statistics

Analysis using type III ANOVA in the mixed model framework identified time as a significant factor (F = 23.43, p < 0.001), indicating that postoperative pain decreased significantly over the follow-up period. The main effect of the treatment group was not statistically significant (F = 2.96, p = 0.091), indicating that overall pain levels did not differ significantly between propolis-treated and control sites. The group × time interaction was also not statistically significant (p = 0.879). Overall, pain scores declined markedly between postoperative days 1 and 7 in both groups, with no statistically significant difference between treatments.

Clinical results

**Figure 10:**
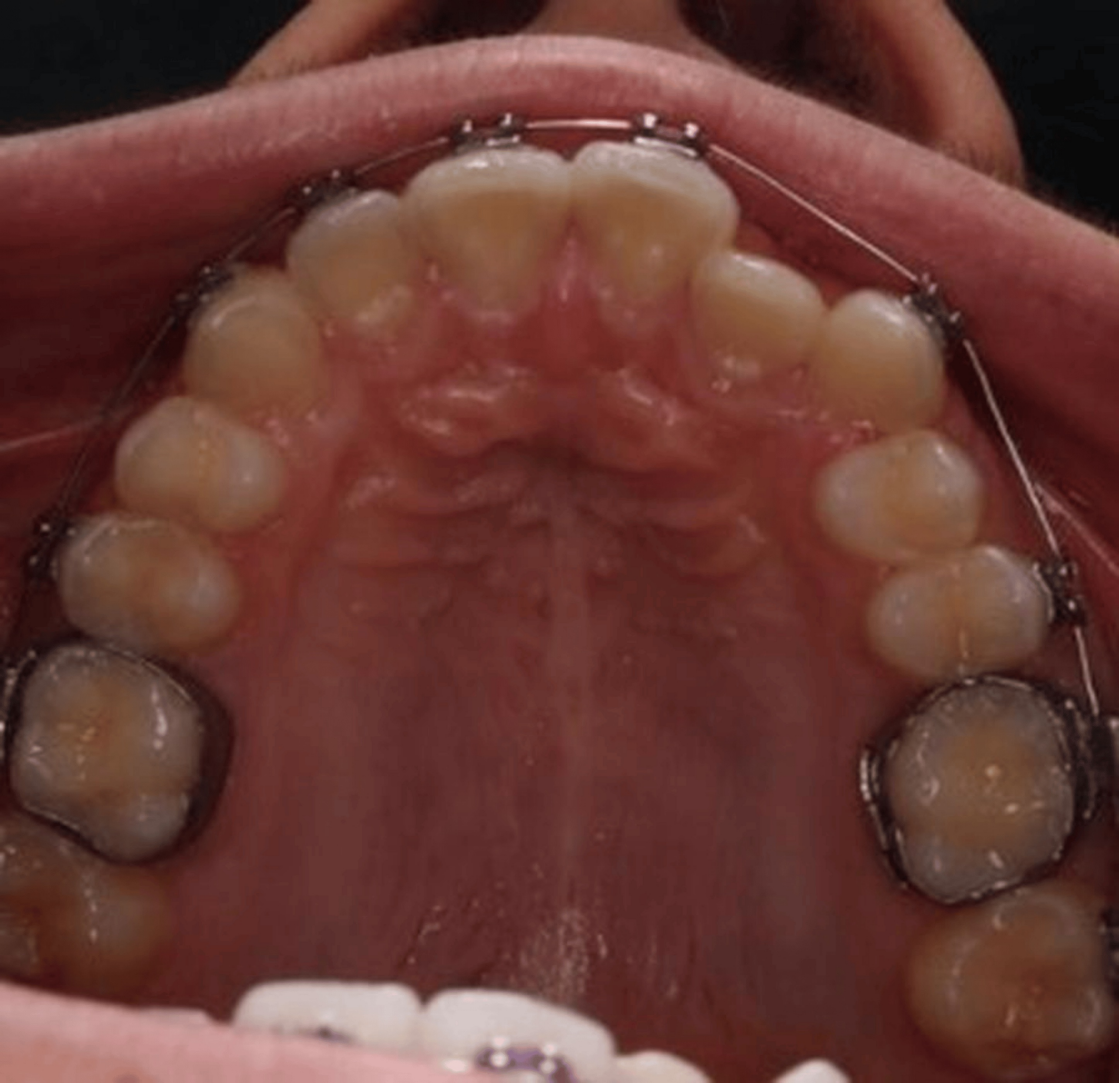
Sites prior to extraction (left site: control; right site: test). Photograph obtained using a Canon EOS R100 digital camera with an RF 100 mm F2.8 L Macro IS USM lens (Canon Inc., Tokyo, Japan).

**Figure 11:**
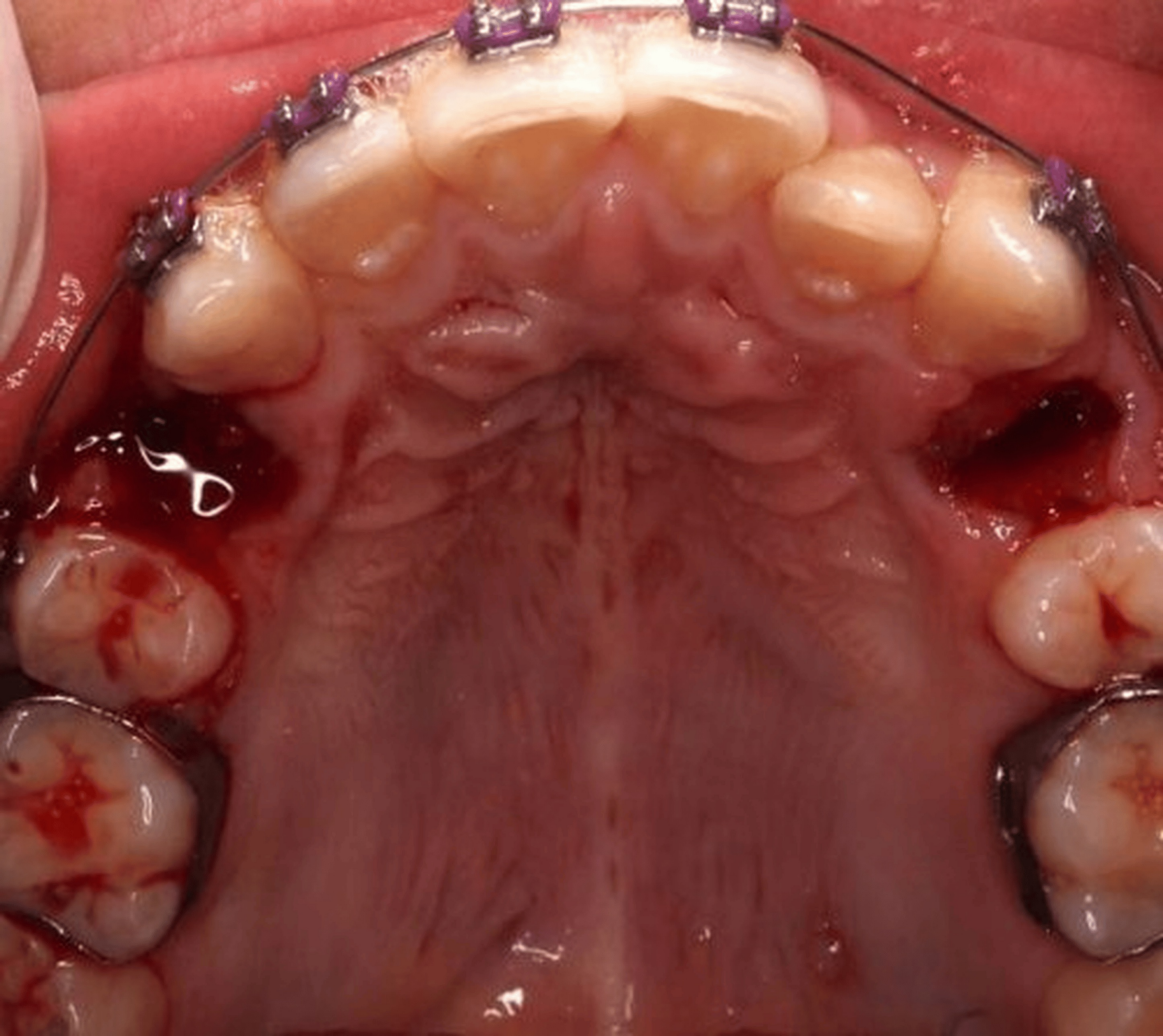
Sites after extraction (left site: control; right site: test). Photograph obtained using a Canon EOS R100 digital camera with an RF 100 mm F2.8 L Macro IS USM lens (Canon Inc., Tokyo, Japan).

**Figure 12:**
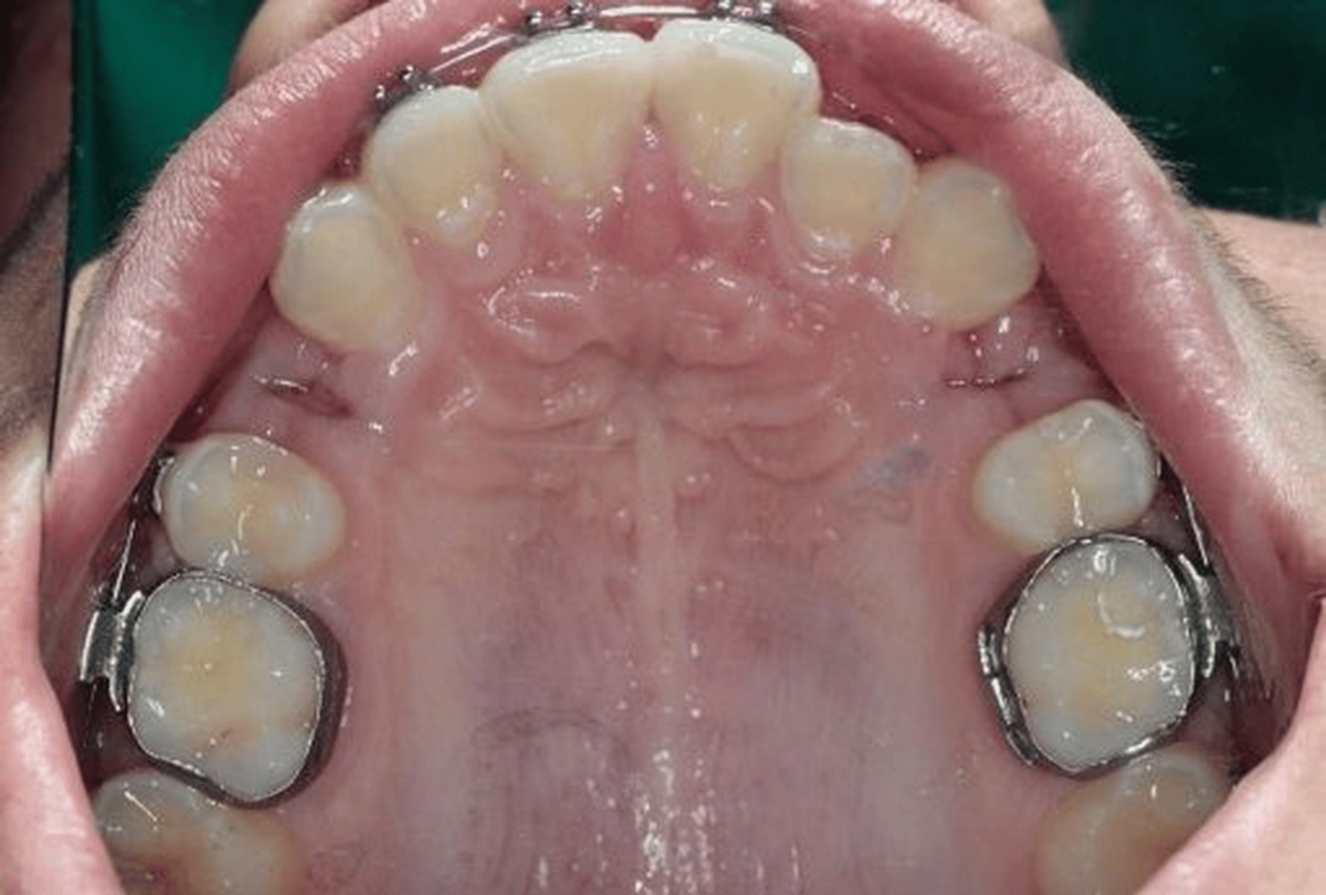
Sites at follow-up day 7 (left site: control; right site: test). Photograph obtained using a Canon EOS R100 digital camera with an RF 100 mm F2.8 L Macro IS USM lens (Canon Inc., Tokyo, Japan).

**Figure 13:**
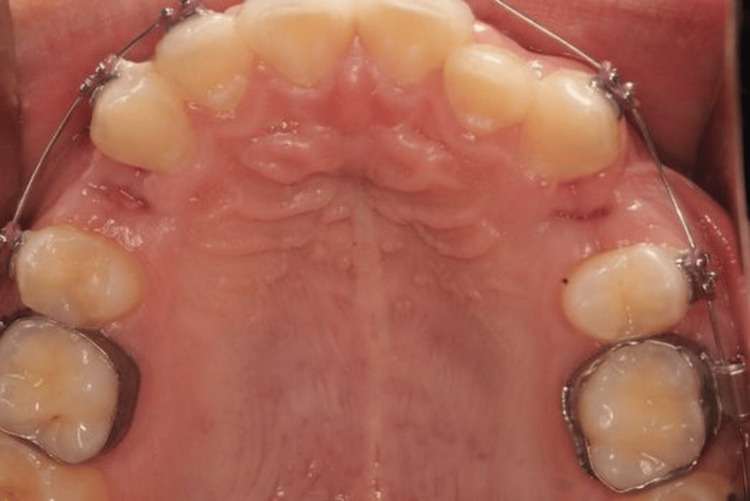
Sites at follow-up day 14 (left site: control; right site: test). Photograph obtained using a Canon EOS R100 digital camera with an RF 100 mm F2.8 L Macro IS USM lens (Canon Inc., Tokyo, Japan).

Bilateral extraction sites are shown prior to the procedure in Figure 10, with the control site on the left and the test site on the right. The appearance of both sockets immediately following extraction is presented in Figure 11. Progressive soft-tissue closure was observed at the day 7 follow-up visit (Figure 12). Further epithelialization was evident at both sites by day 14 (Figure 13).

## Discussion

The present study was designed to evaluate the efficacy of propolis as an adjunctive agent in promoting soft-tissue healing following dental extractions, with a particular focus on early mucosal repair. The results demonstrated that the application of a propolis-based solution significantly enhanced socket healing at both D7 and D14 post extraction, as confirmed through clinical evaluation and three-dimensional measurement analysis. These findings indicate that propolis positively influences the early phases of wound healing, which are critical for optimal tissue regeneration and for minimizing postoperative complications.

Wound healing in the oral cavity is a complex and highly regulated process involving overlapping phases of hemostasis, inflammation, proliferation, and remodeling. Any intervention capable of modulating these phases may significantly improve clinical outcomes. In this context, the observed improvement in healing associated with propolis use suggests that this natural compound may enhance the biological environment of the extraction socket, facilitating faster and more organized tissue repair. However, despite these favorable effects on healing dynamics, the present study did not demonstrate a statistically significant reduction in postoperative pain levels compared to control sites treated with saline alone. This dissociation between improved tissue healing and unchanged pain perception suggests that the mechanisms through which propolis acts are more closely related to cellular regeneration and inflammation control than to direct analgesic effects.

These findings are consistent with previously reported data in the literature. Lisbona-González et al. [11] demonstrated improved clinical healing outcomes following the use of propolis formulations in extraction sockets, supporting its role in enhancing soft-tissue repair. Furthermore, their broader review highlighted the regenerative and biological properties of propolis, while emphasizing the inconsistency of its analgesic effects across studies [12]. Similarly, Askari et al. [13] reported that although propolis may contribute to improved wound healing, its impact on pain reduction remains variable and may depend on factors such as concentration, formulation, and route of administration.

The favorable healing outcomes observed in this study can be explained by the well-established biological properties of propolis. It possesses potent anti-inflammatory, antioxidant, and antimicrobial activities, all of which play a fundamental role in the wound healing cascade [14-17]. By modulating inflammatory mediators such as cytokines and reducing oxidative stress, propolis may create a more favorable microenvironment for tissue repair. Experimental studies have demonstrated that propolis stimulates fibroblast proliferation, enhances collagen synthesis, and promotes angiogenesis, all of which are essential processes during the proliferative phase of healing [18].

Another important consideration is the absence of adverse reactions associated with the use of the propolis solution. No allergic responses were detected during the study period, as confirmed by patch testing, indicating a good safety profile. However, several patients reported experiencing a transient burning sensation immediately following application, which may be attributed to the concentration of active compounds or the presence of ethanol in certain formulations [11]. Despite this mild discomfort, the overall tolerance of the product remained high.

It is also important to consider the potential synergistic contribution of other components present in the Beesline formulation. Beeswax has been reported to exhibit anti-inflammatory properties that may complement the action of propolis and enhance tissue repair [19]. The presence of clove oil (*Eugenia caryophyllata*) in the formulation may have further contributed to the therapeutic effect, as it is widely recognized for its antimicrobial, antifungal, antiviral, antioxidant, and anti-inflammatory properties [20,21] and has been shown to promote mucosal healing [22]. González-Serrano et al. [23] reported that propolis-based formulations significantly reduced postoperative complications such as alveolar osteitis following third molar surgery. Similarly, *Salix alba* (white willow) contains salicin, a precursor of salicylic acid with known anti-inflammatory and analgesic properties [24]. Nevertheless, it is important to acknowledge that the biological activity of propolis is highly variable and depends on factors such as its botanical origin, chemical composition, and method of extraction [25], highlighting the need for standardization of propolis-based products in future research.

Beyond its application in wound healing, propolis has demonstrated a wide range of therapeutic uses in dentistry, including as an intracanal medicament against *Enterococcus faecalis* [26,27] and for inhibiting the growth of *Streptococcus mutans* [28], as well as in propolis-based mouthwashes for reducing dental plaque and gingival inflammation [29].

The sample size was one of the limitations of this study, primarily due to the difficulty in recruiting patients requiring bilateral or symmetrical extractions of similar complexity; the loss of one participant to follow-up further reduced the D14 analysis to 12 patients relative to the 13 available at D7. Another limitation is that although site allocation was randomized via a coin-flip procedure, allocation concealment was not implemented, as the treating operator was aware of the assignment prior to intervention. This lack of concealment may have introduced performance bias, although the impact was likely mitigated by blinded outcome assessment. Additionally, intra-examiner reliability for the repeated surface-area measurements was not formally quantified (e.g., via intraclass correlation coefficient), and formal testing of model assumptions (e.g., normality of residuals, homogeneity of variance) was not performed. The relatively short follow-up period (14 days) precludes conclusions about longer-term healing outcomes. Finally, as the tested formulation combined propolis extract with clove oil and white willow bark, the observed effects cannot be attributed to propolis alone. Results should therefore be interpreted with appropriate caution given the small sample size and exploratory design. Future studies should incorporate concealed allocation methods (e.g., sealed opaque envelopes generated by an independent party), formal reliability metrics, assumption diagnostics, extended follow-up, and isolated single-agent formulations to strengthen internal validity and causal attribution.

## Conclusions

This pilot study suggests that topical application of a propolis-based solution may enhance early soft-tissue closure following dental extraction, without significantly affecting postoperative pain. However, given the small, underpowered sample and the multi-component nature of the tested formulation, these findings should be interpreted as preliminary and hypothesis-generating rather than confirmatory, and the observed benefits cannot be attributed to propolis alone. Larger, adequately powered randomized controlled trials using standardized formulations are warranted to confirm these findings and to establish evidence-based clinical recommendations.

## References

[1] Araújo MG, Lindhe J (2005). Dimensional ridge alterations following tooth extraction. An experimental study in the dog. J Clin Periodontol.

[2] Cardaropoli G, Araújo M, Lindhe J (2003). Dynamics of bone tissue formation in tooth extraction sites. An experimental study in dogs. J Clin Periodontol.

[3] Bosshardt DD, Lang NP (2005). The junctional epithelium: from health to disease. J Dent Res.

[4] Larjava H, Wiebe C, Gallant-Behm C, Hart DA, Heino J, Häkkinen L (2011). Exploring scarless healing of oral soft tissues. J Can Dent Assoc.

[5] Landry RG (1985). Effectiveness of benzydamine HCl in the treatment of periodontal post-surgical patients. Doctoral and Master Theses Prior to ETD Mandate (Pre-2009).

[6] Patzelt SB, Emmanouilidi A, Stampf S, Strub JR, Att W (2014). Accuracy of full-arch scans using intraoral scanners. Clin Oral Investig.

[7] Caso A, Hung LK, Beirne OR (2005). Prevention of alveolar osteitis with chlorhexidine: a meta-analytic review. Oral Surg Oral Med Oral Pathol Oral Radiol Endod.

[8] Koo H, Gomes BP, Rosalen PL, Ambrosano GM, Park YK, Cury JA (2000). In vitro antimicrobial activity of propolis and Arnica montana against oral pathogens. Arch Oral Biol.

[9] Ansorge S, Reinhold D, Lendeckel U (2003). Propolis and some of its constituents down-regulate DNA synthesis and inflammatory cytokine production but induce TGF-β1 production of human immune cells. Z Naturforsch C J Biosci.

[10] Pandis N, Chung B, Scherer RW, Elbourne D, Altman DG (2017). CONSORT 2010 statement: extension checklist for reporting within person randomised trials. BMJ.

[11] Lisbona-González MJ, Muñoz-Soto E, Lisbona-González C, Vallecillo-Rivas M, Diaz-Castro J, Moreno-Fernandez J (2021). Effect of propolis paste and mouthwash formulation on healing after teeth extraction in periodontal disease. Plants (Basel).

[12] Sforcin JM (2016). Biological properties and therapeutic applications of propolis. Phytother Res.

[13] Askari M, Saffarpour A, Purhashemi J, Beyki A (2017). Effect of propolis extract in combination with eugenol-free dressing (Coe-PakTM) on pain and wound healing after Crown-lengthening: a randomized clinical trial. J Dent (Shiraz).

[14] Al-Hatamleh MA, Boer JC, Wilson KL, Plebanski M, Mohamud R, Mustafa MZ (2020). Antioxidant-based medicinal properties of stingless bee products: recent progress and future directions. Biomolecules.

[15] Blonska M, Bronikowska J, Pietsz G, Czuba ZP, Scheller S, Krol W (2004). Effects of ethanol extract of propolis (EEP) and its flavones on inducible gene expression in J774A.1 macrophages. J Ethnopharmacol.

[16] Almuhayawi MS (2020). Propolis as a novel antibacterial agent. Saudi J Biol Sci.

[17] Barroso PR, Lopes-Rocha R, Pereira EM, Marinho SA, de Miranda JL, Lima NL, Verli FD (2012). Effect of propolis on mast cells in wound healing. Inflammopharmacology.

[18] Martinotti S, Ranzato E (2015). Propolis: a new frontier for wound healing?. Burns Trauma.

[19] Carbajal D, Molina V, Valdés S, Arruzazabala ML, Más R, Magraner J (1998). Anti-inflammatory activity of D-002: an active product isolated from beeswax. Prostaglandins Leukot Essent Fatty Acids.

[20] Han X, Parker TL (2017). Anti-inflammatory activity of clove (Eugenia caryophyllata) essential oil in human dermal fibroblasts. Pharm Biol.

[21] Kouidhi B, Zmantar T, Bakhrouf A (2010). Anticariogenic and cytotoxic activity of clove essential oil (Eugenia caryophyllata) against a large number of oral pathogens. Ann Microbiol.

[22] Abd Ressen O, Mahmood FM (2023). The effect of using clove oil on oral mucositis healing among patients undergoing chemotherapy: a randomized control trial. J Genet Environ Conserv.

[23] González-Serrano J, López-Pintor RM, Cecilia-Murga R, Torres J, Hernández G, López-Quiles J (2021). Application of propolis extract, nanovitamin C and nanovitamin E to prevent alveolar osteitis after impacted lower third molar surgery. A randomized, double-blind, split-mouth, pilot study. Med Oral Patol Oral Cir Bucal.

[24] Tawfeek N, Mahmoud MF, Hamdan DI, Sobeh M, Farrag N, Wink M, El-Shazly AM (2021). Phytochemistry, pharmacology and medicinal uses of plants of the genus Salix: an updated review. Front Pharmacol.

[25] Bankova V (2005). Recent trends and important developments in propolis research. Evid Based Complement Alternat Med.

[26] Awawdeh L, Al-Beitawi M, Hammad M (2009). Effectiveness of propolis and calcium hydroxide as a short-term intracanal medicament against Enterococcus faecalis: a laboratory study. Aust Endod J.

[27] Lillygrace E, Kethineni B, Puppala R, Raichurkar HK, Ambati S, Saikiran KV (2021). Antimicrobial efficacy of triple antibiotic paste and propolis as an intracanal medicament in young permanent teeth: an in vivo study. Int J Clin Pediatr Dent.

[28] Hegde KS, Bhat SS, Rao A, Sain S (2013). Effect of propolis on Streptococcus mutans counts: an in vivo study. Int J Clin Pediatr Dent.

[29] Halboub E, Al-Maweri SA, Al-Wesabi M, Al-Kamel A, Shamala A, Al-Sharani A, Koppolu P (2020). Efficacy of propolis-based mouthwashes on dental plaque and gingival inflammation: a systematic review. BMC Oral Health.

